# When the Smear Speaks First: Early Recognition of Thrombotic Microangiopathy in the Absence of Confirmatory Testing

**DOI:** 10.7759/cureus.108833

**Published:** 2026-05-14

**Authors:** Alireza Izadian Bidgoli, Alberto Gomez Veliz

**Affiliations:** 1 Internal Medicine, American University of the Caribbean School of Medicine, Cupecoy, SXM; 2 Internal Medicine, Jackson Memorial Hospital, Miami, USA

**Keywords:** microangiopathic hemolytic anemia, plasma exchange, schistocytes, thrombotic microangiopathy, thrombotic thrombocytopenic purpura

## Abstract

Thrombotic thrombocytopenic purpura (TTP) is a life-threatening thrombotic microangiopathy requiring rapid recognition and prompt treatment. We present the case of a 44-year-old female who was admitted with symptomatic anemia and evolving cytopenias. Laboratory evaluation demonstrated hemolytic anemia with marked reticulocytosis, thrombocytopenia, elevated lactate dehydrogenase, low haptoglobin, and indirect hyperbilirubinemia. Peripheral smear revealed schistocytes, supporting microangiopathic hemolytic anemia. Coagulation studies were largely unremarkable, and direct antiglobulin testing was negative, excluding disseminated intravascular coagulation and autoimmune hemolysis. Extensive imaging, including computed tomography of the brain, chest imaging, echocardiography, and ultrasonography, did not identify an alternative etiology. Given the high clinical suspicion for TTP, empiric plasma exchange therapy was initiated prior to the availability of confirmatory ADAMTS13 testing. The patient demonstrated progressive improvement in platelet count and hemolysis markers with treatment, supporting the diagnosis. This case underscores the importance of early clinical recognition of TTP based on laboratory patterns and peripheral smear findings. Delaying treatment for confirmatory testing may result in significant morbidity and mortality. Prompt initiation of therapy guided by clinical judgment remains essential in suspected cases.

## Introduction

Thrombotic microangiopathies (TMAs) are a group of life-threatening disorders characterized by microangiopathic hemolytic anemia (MAHA), thrombocytopenia, and microvascular thrombosis, leading to end-organ injury [1]. These conditions represent hematologic emergencies requiring rapid recognition and treatment [1]. Thrombotic thrombocytopenic purpura (TTP) is a primary form of TMA caused by severe deficiency of ADAMTS13, resulting in accumulation of ultra-large von Willebrand factor (vWF) multimers and subsequent platelet aggregation within the microcirculation [2].

MAHA is a defining feature of TTP and results from mechanical fragmentation of erythrocytes as they traverse partially occluded small vessels [1-3]. This process produces schistocytes on peripheral blood smear and is accompanied by elevated lactate dehydrogenase, decreased haptoglobin, reticulocytosis, and indirect hyperbilirubinemia, reflecting intravascular hemolysis [1,3]. Although schistocytes are a key diagnostic clue, they are not entirely specific and must be interpreted in the appropriate clinical context [4].

Distinguishing TTP from other causes of TMA, including disseminated intravascular coagulation and secondary TMAs, is essential, as management strategies differ significantly [1]. In particular, relatively preserved coagulation parameters help differentiate TTP from consumptive coagulopathies. Given the high mortality associated with untreated TTP, prompt initiation of plasma exchange therapy is critical and should not be delayed while awaiting confirmatory ADAMTS13 testing [2].

We present a case of suspected TTP in which diagnosis and management were guided by clinical pattern recognition and dynamic laboratory trends, emphasizing the importance of early recognition and timely intervention.

## Case presentation

Clinical presentation 

A 44-year-old female with no known drug allergies was admitted under the hospitalist service for evaluation of symptomatic anemia and systemic illness. The patient's clinical course was notable for progressive fatigue and laboratory abnormalities identified during inpatient evaluation. Over the course of hospitalization, she required close monitoring due to evolving hematologic derangements and concern for a thrombotic microangiopathic process.

Initial assessment and physical exam 

During the early phase of hospitalization, the patient demonstrated worsening anemia accompanied by leukocytosis and fluctuating platelet counts. She developed laboratory evidence suggestive of hemolysis, including rising lactate dehydrogenase levels and indirect hyperbilirubinemia. Her clinical trajectory raised concern for a rapidly evolving hematologic disorder requiring urgent diagnostic clarification and intervention.

Laboratory evaluation demonstrated hemolytic anemia with hemoglobin levels of approximately 8.8-9.9 g/dL and marked reticulocytosis, indicating an appropriate marrow response. Peripheral smear revealed schistocytes with anisocytosis and polychromasia, consistent with MAHA. This was supported by elevated lactate dehydrogenase, low haptoglobin, and indirect hyperbilirubinemia. Platelet counts were reduced early in the course with subsequent recovery following treatment. Leukocytosis with neutrophil predominance was also present. A summary of laboratory results can be found in Table 1. 

**Table 1:** Laboratory findings at admission and during hospitalization. Laboratory values demonstrating hematologic, hemolytic, coagulation, and biochemical parameters at presentation and during the course of hospitalization. Admission values represent initial measurements at presentation, while peak/nadir values reflect the most clinically significant abnormalities or recovery trends observed during treatment. The laboratory profile is notable for severe anemia, thrombocytopenia, elevated markers of hemolysis, and a markedly reduced ADAMTS13 activity, consistent with thrombotic microangiopathy. The relatively preserved coagulation parameters (PT, INR, aPTT, and fibrinogen) argue against disseminated intravascular coagulation. Bleeding time was not performed. Abbreviations: RBC = red blood cell; WBC = white blood cell; MCV = mean corpuscular volume; RDW = red cell distribution width; LDH = lactate dehydrogenase; AST = aspartate aminotransferase; ALT = alanine aminotransferase; BUN = blood urea nitrogen; PT = prothrombin time; INR = international normalized ratio; aPTT = activated partial thromboplastin time; ADAMTS13 = a disintegrin and metalloproteinase with thrombospondin type 1 motif, member 13

Parameter	Admission	Peak/Nadir During Hospitalization	Reference Range (Units)
Hemoglobin	6.5 g/dL	9.9 g/dL	12-16 g/dL
Hematocrit	21.0%	32.2%	36-46%
RBC count	1.97 × 10⁶/µL	2.90 × 10⁶/µL	4.0-5.2 × 10⁶/µL
Platelet count	26 × 10³/µL	343 × 10³/µL	150-400 × 10³/µL
WBC count	13.5 × 10³/µL	18.5 × 10³/µL	4-11 × 10³/µL
MCV	106.6 fL	112.2 fL	80-100 fL
RDW	30.5%	34.1%	11-15%
Reticulocyte %	16.2%	11.1%	0.5-2.5%
LDH	996 U/L	325 U/L	140-280 U/L
Haptoglobin	<20 mg/dL	163 mg/dL	30-200 mg/dL
Total bilirubin	2.7 mg/dL	0.5 mg/dL	0.2-1.2 mg/dL
Direct bilirubin	1.0 mg/dL	0.3 mg/dL	<0.3 mg/dL
AST (SGOT)	90 U/L	36 U/L	10-40 U/L
ALT (SGPT)	161 U/L	71 U/L	7-56 U/L
Creatinine	0.80 mg/dL	0.70 mg/dL	0.6-1.2 mg/dL
BUN	21 mg/dL	15 mg/dL	7-20 mg/dL
Troponin I	0.159 ng/mL	1.6 ng/mL	<0.04 ng/mL
PT (Prothrombin Time)	16.1 sec	14.1 sec	11-13.5 sec
INR	1.27	1.07	0.8-1.1
aPTT	23 sec	25 sec	25-35 sec

Coagulation studies were largely unremarkable, with only mild prolongation of prothrombin time and preserved fibrinogen, arguing against disseminated intravascular coagulation. Renal function remained within normal limits throughout hospitalization, while liver enzymes were moderately elevated. Direct antiglobulin testing was negative, excluding autoimmune hemolysis. Infectious workup, including HIV, hepatitis C, and syphilis testing, was negative, with hepatitis B serology consistent with prior immunization. Urinalysis demonstrated mild proteinuria and hematuria.

Comprehensive imaging was performed to evaluate for secondary causes and complications. Computed tomography of the brain showed no acute intracranial pathology. Chest radiography and additional chest imaging, including GR chest, did not demonstrate pulmonary embolism or acute cardiopulmonary abnormalities. Transthoracic echocardiography revealed no significant structural or functional cardiac abnormalities. Abdominal ultrasound showed no acute hepatobiliary or splenic pathology. Lower extremity venous ultrasound was negative for deep vein thrombosis. Although imaging does not establish the diagnosis of TTP, its consistent negativity across multiple modalities in this case strengthened diagnostic confidence by excluding thromboembolic, structural, and malignant etiologies.

**Figure 1:**
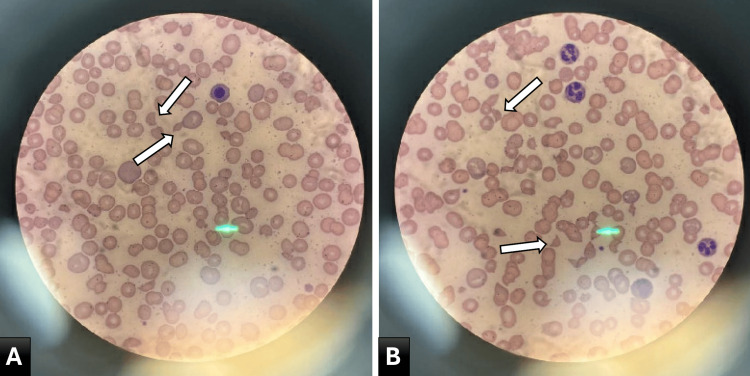
Peripheral blood smear findings. (A) and (B) Peripheral smear demonstrating marked anisopoikilocytosis with numerous schistocytes (arrows), consistent with microangiopathic hemolytic anemia. Occasional polychromasia is noted, reflecting reticulocytosis. These findings support the diagnosis of thrombotic microangiopathy.

ADAMTS13 testing demonstrated severely reduced activity (< 0.10 IU/mL), supporting the diagnosis of TTP. Serial laboratory trends demonstrated progressive improvement in hemolysis markers and platelet count following initiation of TPE, with repeat ADAMTS13 activity improving to 0.58 IU/mL during recovery. Peripheral blood smear demonstrated numerous schistocytes consistent with MAHA (Figure 1).

Multidisciplinary board discussion

Given the constellation of findings, MAHA, thrombocytopenia, elevated LDH, low haptoglobin, and peripheral smear evidence of schistocytes, the case was discussed in a multidisciplinary setting involving hematology, transfusion medicine, and internal medicine teams. The differential diagnosis included TTP, other thrombotic microangiopathies, and, less likely, disseminated intravascular coagulation or severe autoimmune hemolysis. The absence of severe coagulopathy and the presence of classic hemolytic markers favored a diagnosis of TTP or a TTP-like syndrome. Given the high morbidity and mortality associated with delayed treatment, a consensus was reached to initiate empiric therapy.

Diagnosis and management 

A diagnosis of immune-mediated TTP (iTTP) was established based on the presence of MAHA, severe thrombocytopenia, schistocytosis on peripheral smear, biochemical evidence of hemolysis, and confirmed severe ADAMTS13 deficiency (< 0.10 IU/mL). Given the life-threatening nature of the disease, TPE was initiated promptly without delaying treatment for confirmatory testing results.

The patient underwent a total of seven sessions of therapeutic plasmapheresis during hospitalization, with close monitoring of hematologic parameters, renal function, coagulation studies, and markers of hemolysis. Supportive management included transfusion support with fresh frozen plasma and serial laboratory reassessment throughout treatment.

Following initiation of therapy, the patient demonstrated progressive clinical and laboratory improvement, including recovery of platelet count, stabilization of hemoglobin levels, and decline in lactate dehydrogenase and bilirubin levels. Repeat ADAMTS13 activity improved to 0.58 IU/mL during recovery. No worsening renal dysfunction or new neurologic complications developed during treatment.

Clinical outcome 

Following initiation of TPE, the patient completed a total of seven sessions of plasmapheresis, resulting in progressive clinical and laboratory improvement. Hematologic parameters stabilized during the course of treatment, with recovery of platelet count and declining markers of hemolysis, including lactate dehydrogenase and bilirubin levels. Hemoglobin levels gradually improved, and transfusion requirements diminished as the patient's condition stabilized.

Importantly, renal function remained preserved throughout hospitalization, and no neurologic complications developed during treatment. ADAMTS13 activity at presentation was severely reduced (< 0.10 IU/mL), supporting the diagnosis of iTTP.

At the time of discharge, the patient was hemodynamically stable with sustained improvement in laboratory parameters and no evidence of ongoing hemolysis. Given the recognized risk of relapse in iTTP, outpatient hematology follow-up was arranged with continued surveillance of hematologic indices and ADAMTS13 activity as clinically indicated. Overall, the patient's favorable response highlights the importance of early recognition and prompt initiation of plasma exchange in this potentially fatal condition.

## Discussion

Background 

TTP is a rare, life-threatening thrombotic microangiopathy (TMA) first described by Moschcowitz in 1924 in a young woman presenting with anemia, thrombocytopenia, and neurologic symptoms [5]. The condition is characterized by widespread microvascular platelet aggregation due to severe deficiency of ADAMTS13, leading to accumulation of vWF multimers [2].

TTP has an estimated annual incidence of approximately two to six cases per million, with a higher prevalence among women and a peak incidence in adulthood [2,6]. It may occur as an acquired autoimmune disorder or, less commonly, as a congenital condition (Upshaw-Schulman syndrome) [2]. Known risk factors for acquired TTP include autoimmune diseases, infections, pregnancy, certain medications, and malignancy [6].

Clinically, TTP is defined by MAHA and thrombocytopenia, often without a clear anatomical predilection, as microthrombi can affect multiple organ systems, including the brain, kidneys, and heart [6]. It is considered a malignant hematologic emergency due to its rapid progression and high mortality if untreated [7]. Current classifications distinguish TTP within the broader spectrum of thrombotic microangiopathies based on ADAMTS13 activity and clinical presentation [2,7].

Pathology/pathophysiology 

TTP is a disorder of dysregulated hemostasis caused by severe deficiency of the metalloprotease ADAMTS13, which normally cleaves vWF multimers [2]. In acquired TTP, this deficiency is most commonly due to autoantibody-mediated inhibition of ADAMTS13, whereas congenital forms result from mutations in the ADAMTS13 gene [2,6]. The accumulation of ultra-large vWF multimers promotes spontaneous platelet adhesion and aggregation within the microvasculature, leading to widespread formation of platelet-rich thrombi [6].

These microthrombi cause mechanical fragmentation of erythrocytes as they traverse partially occluded vessels, resulting in MAHA characterized by schistocytes on peripheral smear [4]. Concurrent platelet consumption leads to thrombocytopenia, while microvascular occlusion results in tissue ischemia, explaining the potential involvement of multiple organ systems, particularly the brain, kidneys, and heart [6,7].

Histopathologically, TTP is defined by disseminated platelet-rich thrombi within arterioles and capillaries with minimal fibrin deposition, distinguishing it from disseminated intravascular coagulation [7]. This pathophysiologic process directly explains the observed clinical and laboratory findings and supports the use of plasma exchange to remove inhibitory antibodies and restore ADAMTS13 activity.

Comparative analysis with the current literature 

*Clinical Presentation* 

iTTP is classically characterized by MAHA and thrombocytopenia, with variable organ involvement rather than the full historical pentad [8]. The presented case aligns with this modern understanding, demonstrating anemia, thrombocytopenia, schistocytosis, elevated lactate dehydrogenase, and indirect hyperbilirubinemia, which are hallmark laboratory features of MAHA [8,9]. Epidemiologic data indicate that iTTP predominantly affects adults, with a higher incidence in females, consistent with this 44-year-old female patient [9]. However, the absence of significant neurologic impairment and preserved renal function represents relatively milder or atypical features compared with more severe presentations reported in the literature [8,10]. The combination of preserved renal function and the absence of neurologic deficits, while atypical, has been increasingly recognized in early or less severe iTTP presentations, highlighting the spectrum of disease severity.

*Diagnostic Workup* 

The diagnosis of iTTP is challenging because treatment decisions often must be made before confirmatory ADAMTS13 results become available [10]. Current International Society on Thrombosis and Haemostasis (ISTH) guidelines therefore recommend prompt clinical assessment and urgent ADAMTS13 testing without delaying therapy [10]. In this case, schistocytosis on peripheral smear, biochemical evidence of hemolysis, thrombocytopenia, negative direct antiglobulin testing, and a relatively preserved coagulation profile strongly supported iTTP over autoimmune hemolytic anemia or disseminated intravascular coagulation [8,10].

The PLASMIC score is a valuable tool when ADAMTS13 activity is pending, as higher scores correlate strongly with severe ADAMTS13 deficiency and increased likelihood of iTTP [11]. Subsequent confirmation of markedly reduced ADAMTS13 activity (< 0.10 IU/mL) further established the diagnosis and distinguished iTTP from other thrombotic microangiopathies with overlapping features [8,10].

Although imaging studies are not diagnostic for TTP, they are useful in excluding alternative etiologies and complications [8]. In this patient, CT brain, CTA chest, chest radiography, echocardiography, abdominal ultrasound, and lower-extremity venous ultrasound were unrevealing, reinforcing diagnostic confidence in a primary hematologic microangiopathic process. Histopathologic confirmation was not pursued, consistent with modern practice, as TTP is primarily diagnosed using clinical and biochemical criteria rather than tissue biopsy [8,10].

*Management* 

Management of iTTP requires immediate initiation of TPE, which remains the cornerstone of treatment and is strongly associated with improved survival [10]. Because untreated iTTP carries a high mortality risk, current ISTH guidelines recommend initiating therapy based on clinical suspicion rather than delaying treatment pending confirmatory ADAMTS13 results [10]. Early intervention, often within hours of recognition, is considered the most important determinant of outcome [10].

In the present case, plasma exchange was initiated promptly following recognition of MAHA, thrombocytopenia, schistocytosis, and biochemical evidence of hemolysis, consistent with guideline-directed management [8,10]. The patient completed seven sessions of plasmapheresis, with serial laboratory monitoring demonstrating progressive platelet recovery and resolution of hemolysis. Adjunctive immunosuppressive therapy with methylprednisolone was also administered to suppress antibody-mediated inhibition of ADAMTS13, consistent with contemporary treatment strategies for iTTP [10].

Additional therapies increasingly incorporated into modern iTTP management include rituximab and caplacizumab, both of which target the underlying immune-mediated and microthrombotic processes [12]. Rituximab is commonly used in refractory or relapsing disease to reduce autoantibody production, while caplacizumab has been shown to accelerate platelet recovery and reduce disease exacerbation [12]. Although these agents were not utilized in this case, the patient demonstrated a favorable clinical response with plasma exchange and corticosteroid therapy alone.

Overall, the management approach in this case reflects current treatment principles emphasizing rapid recognition, urgent plasma exchange, immunosuppression, and close laboratory surveillance in a potentially fatal but highly treatable hematologic emergency [10].

*Clinical Outcome* 

The patient's improvement in platelet count and hemolysis markers after therapy is consistent with expected response criteria in TTP, where platelet recovery and normalization of hemolysis parameters are central indicators of treatment response [12]. Literature increasingly emphasizes that remission does not end the disease course, because iTTP carries risks of exacerbation, relapse, neurocognitive sequelae, and cardiovascular complications [10-13]. Therefore, adequate follow-up should include monitoring for recurrent thrombocytopenia, hemolysis, ADAMTS13 activity if available, and symptoms suggesting neurologic or cardiac involvement [12,13].

Literature Summary

Compared with recent literature, this case reinforces a central lesson: TTP is a time-sensitive diagnosis in which pattern recognition may be more important than waiting for definitive confirmation [8,10,13]. The case is similar to reported iTTP cohorts in its adult female demographic, MAHA, thrombocytopenia, and response to plasma exchange [8,9]. Its educational value lies in the preserved renal function, negative broad imaging workup, and reliance on serial laboratory trends to support management. This case reinforces that, in suspected TTP, diagnostic certainty is often retrospective, while therapeutic urgency must be immediate.

What we learned from this case 

This case highlights the critical importance of recognizing TMA through pattern recognition rather than reliance on a single confirmatory test. The combination of hemolytic anemia, thrombocytopenia, elevated lactate dehydrogenase, low haptoglobin, and schistocytes on peripheral smear formed a diagnostic constellation that was sufficient to justify early empiric treatment for TTP, even in the absence of immediately available ADAMTS13 activity results. This reinforces the principle that TTP remains a clinical diagnosis in its early phase, where delays in therapy can significantly increase mortality.

A key learning point is the diagnostic value of a normal coagulation profile in distinguishing TMA from disseminated intravascular coagulation. In this case, preserved fibrinogen levels and only minimal prolongation of prothrombin time helped narrow the differential and supported a non-consumptive process. Similarly, the negative direct antiglobulin test effectively excluded autoimmune hemolysis, emphasizing the importance of systematically ruling out competing etiologies.

Another important insight is the role of comprehensive but targeted evaluation. Extensive imaging, including CT brain, CTA chest, echocardiography, and ultrasound studies, did not reveal alternative pathology, thereby strengthening diagnostic confidence in a hematologic process rather than a structural or thromboembolic cause. This underscores how negative imaging findings, when appropriately contextualized, can be diagnostically valuable rather than merely ancillary.

Finally, this case illustrates the utility of serial laboratory trends over isolated values. The dynamic improvement in platelet count and decline in hemolysis markers following intervention provided both diagnostic confirmation and a real-time measure of therapeutic response. This reinforces the concept that, in suspected TTP, early treatment should not be deferred for confirmatory testing, and the clinical trajectory itself becomes part of the diagnostic process.

Collectively, this case emphasizes a pragmatic, physiology-driven approach: prioritize early recognition of TMA patterns, initiate treatment promptly, and use evolving clinical and laboratory data to refine and confirm the diagnosis.

## Conclusions

This case underscores the critical importance of early recognition and prompt treatment of TTP as a clinical diagnosis. In the setting of MAHA and thrombocytopenia, supported by characteristic laboratory findings and peripheral smear abnormalities, immediate initiation of plasma exchange should not be delayed while awaiting confirmatory testing, such as ADAMTS13 activity. The absence of significant coagulation abnormalities and a negative direct antiglobulin test are key elements in narrowing the differential diagnosis and distinguishing TTP from other causes of hemolysis.

Equally important is the role of serial laboratory monitoring, which not only supports the initial diagnosis but also provides a real-time assessment of therapeutic response. This case highlights how a structured, physiology-driven approach, integrating laboratory patterns, exclusion of competing diagnoses, and timely intervention, can lead to favorable outcomes in a potentially fatal condition. Early clinical judgment remains the cornerstone of effective management in suspected TTP.
